# Collective Strategy Condensation: When Envy Splits Societies

**DOI:** 10.3390/e23020157

**Published:** 2021-01-27

**Authors:** Claudius Gros

**Affiliations:** Institute for Theoretical Physics, Goethe University Frankfurt, 60323 Frankfurt, Germany; gros07@itp.uni-frankfurt.de

**Keywords:** self-organization, sociophysics, game theory, strategy condensation, nash equilibrium, phase transition, envy, social classes, complex systems

## Abstract

Human societies are characterized by three constituent features, besides others. (A) Options, as for jobs and societal positions, differ with respect to their associated monetary and non-monetary payoffs. (B) Competition leads to reduced payoffs when individuals compete for the same option as others. (C) People care about how they are doing relatively to others. The latter trait—the propensity to compare one’s own success with that of others—expresses itself as envy. It is shown that the combination of (A)–(C) leads to spontaneous class stratification. Societies of agents split endogenously into two social classes, an upper and a lower class, when envy becomes relevant. A comprehensive analysis of the Nash equilibria characterizing a basic reference game is presented. Class separation is due to the condensation of the strategies of lower-class agents, which play an identical mixed strategy. Upper-class agents do not condense, following individualist pure strategies. The model and results are size-consistent, holding for arbitrary large numbers of agents and options. Analytic results are confirmed by extensive numerical simulations. An analogy to interacting confined classical particles is discussed.

## 1. Introduction

The notion of an “ideal society” has always been controversial [1,2], especially regarding the conditions for social classes to arise endogenously when by-birth privileges and handicaps are absent, a feature commonly presumed to be desirable. In this regard, one may consider a society to be “ideal” when the playing ground is fair, which means that members have equal access to societal options and positions. Here, we examine this situation using a generalized game theoretical setting.

Two building blocks constitute the core of most abstract games [3]: competition, and that different options yield distinct rewards. In this study, we examine what happens if a third element is added, postulating that agents desire to compare their individual success reciprocally, a trait usually termed “envy” [4]. We show that envy splits ideal societies. Two distinct social classes, an upper and a lower class, form endogenously when the desire to compare success becomes substantial.

The notion of envy is based on the observation that the satisfaction individuals receive from having and spending money depends not only on the absolute level of consumption, but also on how one’s own consumption level compares with that of others [5]. This view, which is at the heart of relative income theory [6,7], is taken for granted, to give an example, when poverty is defined not in absolute, but in relative terms [8,9].

The key to our research is the notion that class structures may emerge from class-neutral interactions between individual agents. On an equivalent basis, a large body of social computation research [10,11] has investigated to which extent cooperation [12,13], reciprocity [14], altruism [15], and social norms [16] are emergent phenomena. The model investigated in this study is formulated directly in terms of strategies, as usual for animal conflict models [17], like the war of attrition. A corresponding agent-based simulation setup would also be possible, with the differences vanishing in the limit of large numbers of agents and behavioral options. The adaptive game-theoretical formulation used here comes with the advantage that the properties of the class-stratified state can be studied analytically. Our study can be seen as a generalization of evolutionary game theory, which is dedicated in good part to the origins of behavioral traits [18], and to the emergence of class structures. Other alternatives include dynamical system investigations of the stability of societies [19], and game theoretical approaches centered on selected key societal players [20].

### Social Classes in Terms of Reward Clusters

We consider a society of *N* agents, with every agent able to select from *M* options. The payoff function $E_{i}^{\alpha }$, for option *i* and agent α, is agent-specific, but only to the extent that it depends explicitly on the strategies $p_{i}^{\alpha }\geq 0$. Strategies are normalized, $\sum_{i}p_{i}^{\alpha }=1$, with $p_{i}^{\alpha }\geq 0$ denoting the probability that agent α selects option *i*. Rewards $R^{\alpha }$ are defined as the expected payoffs,

(1) $$R^{\alpha }=\sum_{i}E_{i}^{\alpha }p_{i}^{\alpha }={\langle E_{i}^{\alpha }\rangle }_{\left\{\rho_{i}^{\alpha }\right\}}\;.$$

The number of options *M* can be both larger or smaller than the number of agents *N*, with size-consistent large-*N* limits being recovered for constant ratios ν=M/N.

**Figure 1:**
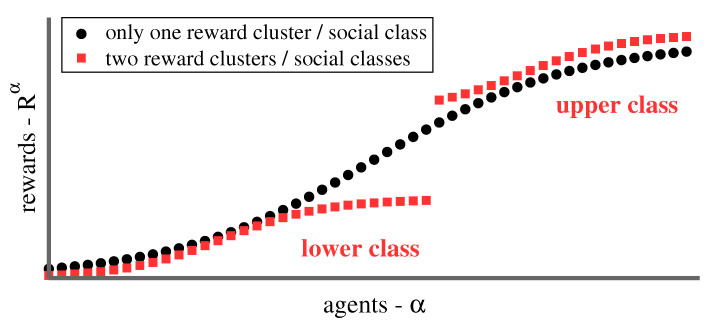
Social classes as reward clusters. When ordering the rewards $R^{\alpha }$ obtained by agents α as a function of size, the resulting reward spectrum may be characterized either by a continuous distribution (black), or by one or more gaps (red). Clustering rewards with regard to proximity consequently allows for a bare-bone definition of social classes.

In this study, we define social classes in terms of reward clusters. Agents within the same class receive rewards similar in magnitude, which are separated by a gap from the rewards of other classes, as illustrated in Figure 1. In human societies, social groups are also particularly shaped by the notion of social identity [21], which is absent in the bare-bone definition of social classes used here. A political theory for social classes is beyond the scope of the present study.

## 2. Shopping Trouble Model

We define with

(2) $$\bar{R}=\frac{1}{M}\sum_{\alpha }R^{\alpha }\;,$$

the mean reward $\bar{R}$ of all agents. The payoff function

(3) $$E_{i}^{\alpha }=v_{i}-\kappa \sum_{\beta \neq \alpha }p_{i}^{\beta }+\varepsilon \;p_{i}^{\alpha }\log\left(\frac{R^{\alpha }}{\bar{R}}\right)$$

of our reference model contains three terms:

- **Basic utility.** The basic utility function $v_{i}$, which is identical for all agents, encodes the notion that options come with different payoffs. Mapping options to qualities $q_{i}\;\in \;\left[0,2\right]$, we will use a simple inverted parabola, $v_{i}=1-{\left(1-q_{i}\right)}^{2}$, for the basic utility.
- **Competition.** There is a flat penalty κ for agents competing heads-on. Payoff reduction is proportional to the probability $p_{i}^{\beta }$ that other agents select the option in question. The respective combinatorial factors are approximated linearly in (3), as given by the sum $\sum_{\beta \neq \alpha }p_{i}^{\beta }$.
- **Envy.** One’s own success with respect to the mean reward, $R^{\alpha }/\bar{R}$, induces a psychological reward component.

The log-dependency $\log(R^{\alpha }/\bar{R})$ of the envy term in (3) reflects the well-established observation that the brain discounts sensory stimuli [22], numbers [23], time [24], and data sizes [25] logarithmically. In addition, the envy term is proportional to the current probability $p_{i}^{\alpha }$ to select option *i*, which encodes a straightforward rational. When everything is fine, when $\log(R^{\alpha }/\bar{R})\;>\;0$, the current strategy is reinforced, and suppressed when $\log(R^{\alpha }/\bar{R})\;<\;0$. The effect is that agents with high/low rewards tend to pursue pure/mixed strategies. Equation (3) is called the “shopping trouble model”, as it can be applied, besides the general social context, to the case that agents need to optimize their shopping list [26]. Note that that agents have only a single goal within the shopping trouble game—reward maximisation—in contrast to most status-seeking games [27,28], for which both status and utility are separately important [29,30].

### 2.1. Correspondence to Confined Interacting Classical Particles

**Figure 2:**
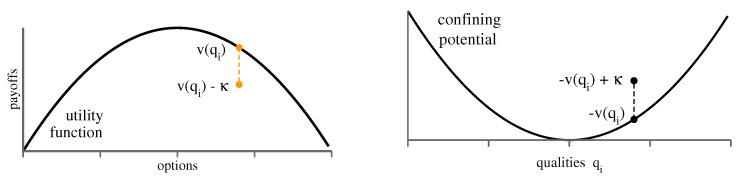
Correspondence to interacting classical particles. (**Left**) Agents selecting a strategy *i* receive a bar utility $v(q_{i})$ (inverted parabola), which is reduced by a flat amount κ, the competition term, if another agent selects the same option. Utilities are to be maximized. (**Right**) Classical particles in a confining potential $-v(q_{i})$ (parabola) repel each other by an amount −κ. Energy is minimized.

The shopping trouble model (3) can be interpreted in terms of interacting and confined classical particles, with the correspondence
agents↔classical particles$q_{i}$↔states$-v(q_{i})$↔confining potentialκ↔Coulomb repulsionϵ↔energy-dependent interaction
as is illustrated in Figure 2. Without the energy-dependent interaction term, ϵ (envy), particles settle into the respective lowest energy states, which are given by $-v\left(q_{i}\right)+\left(n_{i}-1\right)\kappa$, where $n_{i}$ is the occupation number of state $q_{i}$ (the number of agents selecting the quality $q_{i}$). Strategies $p_{i}^{\alpha }$ correspond in physics terms to the occupation distribution. At finite temperatures, particles can always swap places, which implies that strategies are identical. However, this is not the case at zero temperature. Finite temperatures correspond in game theory that agents select alternative, lower-reward strategies, with a probability given by the respective Boltzmann factors. Here, we work with strictly rational, zero-temperature agents, which always go for the best choice.

### 2.2. Strategy Evolution

Agents interact in the shopping trouble model via two averaging fields [31]. The first coupling term is a scalar quantity, the average reward $\bar{R}$. It quantifies the envy term in (3). The second coupling term, the mean strategy ${\bar{p}}_{i}=\sum_{\beta }p_{i}^{\beta }/M$, is in contrast to a function of the available options. It enters the penalty term via

(4) $$\sum_{\beta \neq \alpha }p_{i}^{\beta }=M{\bar{p}}_{i}-p_{i}^{\alpha }\;.$$

Numerically, the shopping trouble model is solved using standard evolutionary dynamics [32],

(5) $$p_{i}^{\alpha }\left(t+1\right)=\frac{p_{i}^{\alpha }\left(t\right)E_{i}^{\alpha }\left(t\right)}{\sum_{j}p_{j}^{\alpha }\left(t\right)E_{j}^{\alpha }\left(t\right)}\;.$$

In practice, a constant offset $E_{0}$ is added on the right-hand side, which acts as a smoothing factor.

### 2.3. Pure vs. Mixed Strategies

**Figure 3:**
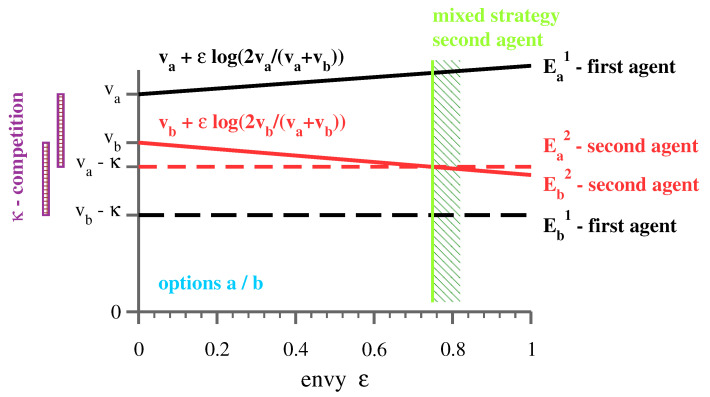
Envy-induced transition from pure to mixed strategies. Illustration of the case of two agents that can select between two options, a/b, with basic utilities $v_{a}$ and $v_{b}$. Here, $v_{a}>v_{b}$. In the absence of envy, ϵ=0, both agents play pure strategies, here with the first/second agent selecting a/b. It would be unfavorable for the second agent to invade option *a*, as $v_{a}-\kappa <v_{b}$, and vice versa, where κ is the strength of the competition. In this state, rewards are $R_{a}=v_{a}$ and $R_{b}=v_{b}$ and $R_{a,b}/\bar{R}=2v_{a,b}/\left(v_{a}+v_{b}\right)$. For the second agent, the envy term $\epsilon p_{i}^{\alpha }\log\left(R^{\alpha }/\bar{R}\right)$ is negative for the *b*-option, vanishing for the *a*-option. The second agent starts to play a mixed strategy (green shaded area) when the payoff $E_{b}^{2}=v_{b}+\epsilon \log\left(2v_{b}/\left(v_{a}+v_{b}\right)\right)$ (red solid line) becomes smaller than the $E_{a}^{2}=v_{a}-\kappa$ (red dashed line).

The support of a strategy $p_{i}^{\alpha }$ is given by the set of options selected with finite probabilities $p_{i}^{\alpha }>0$. The smallest possible support is one, the case of a pure strategy, $p_{i}^{\alpha }=\delta_{i,k}$. Supports larger than one correspond to mixed strategies. Without envy, viz when ϵ=0, the Nash stable strategies of the shopping troubling game are all pure. Agents just compare the payoff options $v_{i}-\kappa \left(n_{i}-1\right)$ of distinct options, where $n_{i}$ is the occupation factor, viz the number of times option *i* has been selected by all agents. If not favorable, agents will avoid occupied options and settle for lower basic utilities. The situation is illustrated for two players in Figure 3. By avoiding each other, agents seemingly cooperate, a state called “forced cooperation” [26].

Relative payoff magnitudes change when envy is introduced. The own option becomes progressively less attractive when the envy term is negative, which is the case for agents below $\bar{R}$, see Figure 3. Eventually, the payoff for the own option levels with that of an occupied option with a higher basic utility and mixed strategies appear. For larger numbers of agents and options, we find that the evolution of mixed strategies with increasing levels of envy leads to a class-stratified state, as discussed further below.

## 3. Results

**Figure 4:**
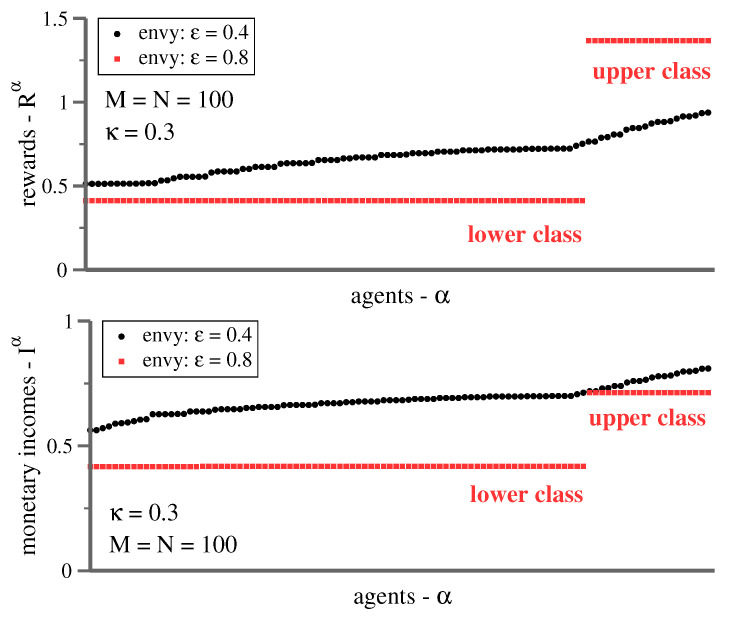
Envy-induced class stratification. Simulation results for M=N=100 and κ=0.3. (**Top**) For ϵ=0.4 (black) the reward spectrum is continuous, with agents receiving varying rewards. For ϵ=0.8 (red) two strictly separated reward clusters emerge. Members of the same class receive identical rewards, which implies intra-class communism. (**Bottom**) The respective spectrum of monetary incomes $I^{\alpha }$, as defined by Equation (6). The gap between the lower and upper classes is substantial. Note that everybody’s monetary income drops when envy is increased from 0.4 to 0.8. Percentage-wise, the loss is comparatively small for top-income agents.

In Figure 4, representative reward distributions for the shopping trouble model are given. The results are obtained iterating (5) recursively for $5\;\times \;10^{5}$ times. The initial strategies are random, which implies that chance determines the fate of individual agents, particularly the final reward. Equivalent results are obtained for smaller and larger numbers of agents and options. Changing the density of agents per option, ν=M/N, leads to quantitative, but not qualitative changes. Larger values of ν increase the influence of competition, κ, and hence, also of envy. The same holds when increasing κ directly.

The transition from forced cooperation at κ=0.4 to class separation, for κ=0.8, observed in Figure 4 induces a striking self-organized reorganization of the reward spectrum. The distribution of rewards is continuous, but otherwise inconspicuous below the transition. A finite competition of κ=0.3 induces cooperation in the sense that it is generally favorable for agents to select different options. Two flat bands arise in contrast in the class-stratified state, one for the upper and one for the lower class.

**Figure 5:**
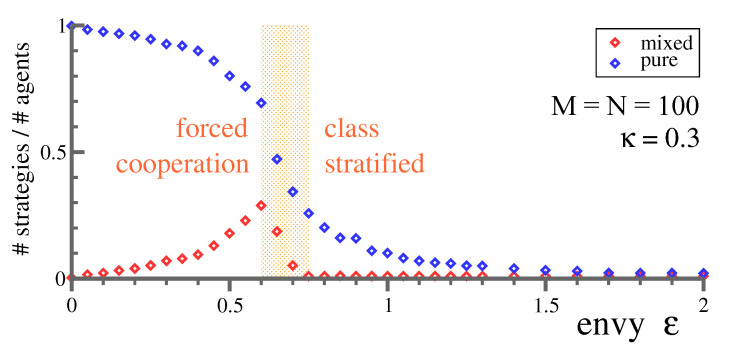
Evolution of mixed strategies. For N=100 options and M=100 agents, the fraction of agents playing pure and mixed options respectively. For small envy, the number of mixed strategies rises, in agreement with the mechanism illustrated in Figure 3 for the case of two players. Mixed strategies played by distinct agents merge into a single mixed strategy for the entirety of lower-class agents once a critical density of mixed strategies is reached. The shaded region denotes bistability. When starting from random initial strategies and values of ϵ in the shaded region, the evolutionary dynamics (5) lead to either of two possible Nash equilibria, forced cooperation, and class stratification. The fraction of pure strategies drops for all ϵ, until only one or two upper-class members remain, the monarchy state. Adapted from [26].

The observation that all lower-class agents receive identical rewards has a relatively simple explanation. The number of mixed strategies first rises with ϵ, in order to drop to one in the class-stratified state. Compare Figure 5. Inspecting the individual strategies one-by-one reveals that an identical mixed strategy is played by the entirety of lower-class agents. Colloquially speaking, one becomes a member of the masses when joining the lower class. This result explains that a single mixed strategy remains in the class stratified-state, and that all members of the lower class receive the same reward.

**Figure 6:**
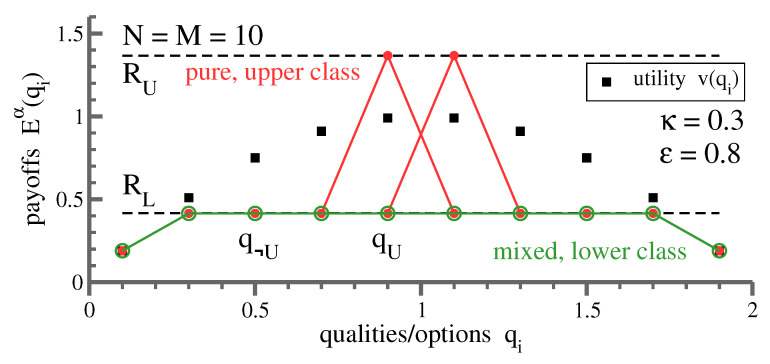
Payoffs in the class-stratified state. Numerically obtained payoff functions $E_{i}^{\alpha }=E^{\alpha }\left(q_{i}\right)$, for a system with 10 options/agents. The strength of competition/envy is κ=0.3 and ϵ=0.8. Shown is the payoff function for the two pure upper-class strategies (red), and for the single mixed lower-class strategy (green), played by eight agents. For the functional form of the bare utility, $v_{i}=v\left(q_{i}\right)$, an inverse parabola has been selected (black squares). Also shown are the analytic expressions (8) and (7) for the upper-/lower-class rewards, $R_{U}$ and $R_{L}$ (dashed horizontal lines). Indicated by $q_{U}$ and $q_{\neg U}$ are qualities played/not played by the upper class.

In contrast to the lower class, upper-class agents play pure strategies. Members of the upper class avoid each other, and their strategies are hence individualistic, as illustrated in Figure 6 for a small system. Why is it then, as evident from Figure 4, that upper-class agents have identical rewards? This effect is due to the interaction with the lower-class mixed strategy, which adapts itself autonomously until the contribution from competition—the term ∼κ in (3)—exactly cancels out the reward differential arising from differences in the respective basic utilities, $v_{i}$. One can trace analytically, as discussed further below, why this remarkable self-organized process takes place.

### 3.1. Monetary Incomes—Everybody Loses

It is evident from the top panel of Figure 4 that envy induces the formation of two well-defined reward clusters. The question arises whether the gap between the lower- and the upper-class clusters is purely psychological, viz exclusively due to the envy term in (3). For this purpose, we define with

(6) $$I^{\alpha }=\sum_{i}\left(v_{i}-\kappa \sum_{\beta \neq \alpha }p_{i}^{\beta }\right)p_{i}^{\alpha }$$

the monetary income $I^{\alpha }$, which represents the reward $R^{\alpha }$ minus the envy contribution. Figure 4 shows that a gap opens, both for the reward and for the monetary income. Everybody loses when envy increases, in the sense that monetary income drops for all agents, also for those at the top, when increasing levels of envy force the society to class-separate.

### 3.2. Analytic Properties of the Class-Stratified State

The agent-to-agent interaction is mediated in the shopping trouble model by two averaging fields, $\bar{R}$ and ${\bar{p}}_{i}$, as discussed further above. It can be shown [26] that this property allows to derive analytic expressions for the rewards of the lower and of the upper class, respectively, $R_{L}$ and $R_{U}$,

(7) $$R_{L}=\varepsilon \;\frac{1-f_{L}}{e^{\kappa /\varepsilon }-1}\log\left(\frac{e^{\kappa /\varepsilon }-f_{L}}{1-f_{L}}\right)$$

and

(8) $$R_{U}=\varepsilon \;\frac{1-f_{L}e^{-\kappa /\varepsilon }}{1-e^{-\kappa /\varepsilon }}\log\left(\frac{e^{\kappa /\varepsilon }-f_{L}}{1-f_{L}}\right)\;.$$

Remarkably, the above expressions are not explicitly dependent on the basic utility $v_{i}$. The only free parameter in (7) and (8) is the fraction $f_{L}$ of agents in the lower class, which can be determined numerically. For the class-stratified state shown in Figure 4, one has, as an example, $f_{L}=80/100=0.8$, as the number of lower- and upper-class agents is 80 and 20, respectively. Using $f_{L}=0.8$ in (7) and (8), the resulting values for $R_{L}$ and $R_{U}$ coincide exactly with the values obtained numerically.

### 3.3. Monarchy vs. Communism

The continuous downsizing of the upper class observable in Figure 5 raises an interesting question. Is there a critical envy ϵ, beyond which the upper class vanishes altogether? In this case, agents would exclusively play the mixed strategy of the former lower class, a telltale characteristic of a communist state. Rewards would also be the same for everybody. This hypothesis can be tested numerically by using the extracted lower-class mixed strategy as the starting strategy for all *M* agents. Even for large ϵ, we performed simulations up to ϵ=20, where the communist state is found to be numerically unstable. The system converges without exception to a class-separated state containing one or two upper-class agents. Within the shopping trouble model, communism is unstable against monarchy.

## 4. Terminology

The notation used throughout this study is summarized below. The aim of the compendium below is to provide an overview, not complete and detailed definitions.

**Options, qualities, and strategies.** Options correspond to possible actions, such as making a purchase in a shop. The numerical value associated with option *i* is the quality $q_{i}$. Furthermore, we differentiate between option and strategy, which is defined here as the probability distribution function $p_{i}=p\left(q_{i}\right)$ to pursue a given option.

**Pure vs. mixed strategies.** A strategy is pure when the agent plays the identical option at all times, and mixed otherwise, viz when behavior is variable.

**Evolutionary stable strategies.** Taking the average payoff received as an indicator for fitness, a given strategy is evolutionary stable if every alternative leads to lower fitness. Evolutionarily stable strategies are Nash-stable.

**Support.** Strategies are positive definite for all options, $p^{\alpha }\left(q_{i}\right)\geq 0$. In reality, $p^{\alpha }\left(q_{j}\right)$ is finite only for a subset of options, the support of the strategy. Strategies are pure/mixed when the size of the support is one/larger than one.

**Payoff/reward.** The payoff function is a real-valued function of the qualities (options). The mean payoff, as averaged over the current strategy, is the reward.

**Competitive/cooperative game.** Parties may coordinate their strategies in cooperative games, but not in competitive games. For the shopping trouble game, voluntary cooperation is not possible.

**Collective effects/phase transition.** The state of a complex system, like a society of agents, may change qualitatively upon changing a parameter, f.i. the strength of envy. Such a transition corresponds in physics terms to a phase transition. Phase transitions are in general due to collective effects, which means that they are the result of the interaction between the components, here the agents.

**Forced cooperation/class stratification.** Forced cooperation is present when agents seemingly cooperate by avoiding each other, as far as possible. It is forced when, in reality, agents optimize just their individual fitness. Forced cooperation and the class-stratified state are separated by a collective phase transition.

**Envy.** Envy is postulated to have opposite effects on agents with high/low rewards. When their reward is above the average, agents take this as an indication that they are doing well and that the best course of action is to enhance the current strategy. In contrast, agents with below-average rewards are motivated to search for alternatives, viz to change the current strategy.

**Monarchy and communism.** Monarchy and communism are used throughout this study exclusively for the labeling of states defined by specific constellations of strategies. Secondary characterizations in terms of political theory are not implied. Monarchy is present in a class-stratified society when all but one or two agents belong to the lower class. All members of the society are part of a unique class in communism, with everybody receiving identical rewards and following the same mixed strategy.

## 5. Discussion

The payoff function of the shopping trouble model (3) is not static, as in classical games, but highly adaptive. The payoff received when selecting a certain action *i* depends dynamically on the strategies of the other agents. At its core, this is typical for social simulation studies [10], with the twist that the shopping trouble model is formulated directly in terms of strategies. The resulting evolutionary stable strategies are hence to be determined self-consistently, which implies that certain aspects of the multi-agent Nash solutions may have emergent character [33]. This has indeed been observed.

The shopping trouble model studied here incorporates specific functionalities. We believe, however, that alternative models based on the same three principles, payoff diversity, competition, and inter-agent reward comparison, would lead to qualitatively similar results. Further, we note that the widely used distinction between benign and malicious envy [34] enters the shopping trouble model, albeit indirectly. Benign envy, the quest to reach a better outcome by improving oneself, can be said to be operative when agents select the best pure strategy compatible with everybody else’s choices. Malicious envy, which aims to pull somebody down from their superior position [34], is functionally operative when agents start to invade somebody else’s zone by switching to mixed strategies, as shown in Figure 3. In this interpretation, societies are pushed towards class stratification by malicious, and not by benign envy. On a societal level, malicious envy is counterproductive.

## 6. Conclusions

We conclude by recapitulating the driving forces for the class stratification transition. Agents with low rewards are constantly searching for better options (compare Section 4). However, more than one option can be sampled only when using mixed strategies, which implies that raising levels of envy induce a corresponding larger number of mixed strategies, as observed in Figure 5. In the end, a large number of low-reward agents are trying to explore an extended range of options. At a certain level of envy, their respective mixed strategies collide, collapsing at this point into a single encompassing strategy for the entire lower class. Class stratification is hence a result of a spontaneous condensation of strategies.

In effect, class stratification results from the constant state of discontent of low-reward agents, taking place right at the point when their continuing search for alternatives runs out of options. In contrast, high-reward agents have little incentive to do anything else. In order to keep their privileged position, they just need to concentrate their efforts on what they do best—that is, their current strategies.
